# Bacterial Contamination in Dental Unit Water Lines at Primary Health Care Centers (2022–2023): A Nationwide Study

**DOI:** 10.3390/ijerph22091406

**Published:** 2025-09-09

**Authors:** Abrar Jamal, Eiman Alawadhi

**Affiliations:** 1 Department of Preventive Medicine, Public Health Administration, Ministry of Health, Kuwait City 43610, Kuwait; abrar211124887@ku.edu.kw; 2 Department of Epidemiology and Biostatistics, College of Public Health, Kuwait University, Kuwait City 13110, Kuwait

**Keywords:** dental unit waterlines, bacterial contamination, disinfection, water quality

## Abstract

**Background:** Dental unit water lines (DUWLs) can harbor microbial contamination, posing risks for cross-infection to patients and dental staff. This study assessed the prevalence of bacterial contamination in DUWLs at primary healthcare centers in Kuwait during 2022–2023 and examined variation by year, governorate, and sampling outlet. **Methods:** A retrospective cross-sectional analysis was conducted using 3290 water test results from six governorates. Data were obtained from the Environmental Health Department, Ministry of Health, and analyzed using STATA 17. Contamination was defined as a total plate count (TPC) > 100 CFU/mL or presence of coliforms, *Escherichia coli*, *Pseudomonas aeruginosa*, or fecal streptococci. Descriptive and logistic regression analyses were performed. **Results:** Overall contamination prevalence was 16.8%. Rates were higher in 2023 (19.8%) than in 2022 (13.7%) and higher in Mubarak Alkabeer governorate (23%) and cup filler outlets (18.9%). Logistic regression showed significantly increased odds of contamination in 2023 (OR = 1.6; 95% CI: 1.3–2.0), Mubarak Alkabeer (OR = 1.4; 95% CI: 1.1–1.9), and cup fillers (OR = 1.3; 95% CI: 1.1–1.6). *P. aeruginosa* was detected in 1.3% of samples. **Conclusions:** One in six DUWL samples exceeded Kuwait’s strict microbial safety threshold. Findings highlight spatial and procedural variations in contamination and underscore the need for enhanced disinfection protocols, preventive maintenance, and targeted staff training to ensure waterline safety.

## 1. Introduction

Dental Unit Water Lines (DUWLs) are essential for daily dental procedures but are known reservoirs for microbial contamination due to long tubing, low flow rates, and prolonged water stagnation [1,2]. These conditions facilitate biofilm formation—surface-attached bacterial colonies encased in a protective matrix, which can harbor opportunistic pathogens, such as *Pseudomonas aeruginosa*, *Escherichia coli*, fecal streptococci, *Legionella* spp., and nontuberculous mycobacteria, posing risks to patients and dental personnel [3,4]. Water sources for dental units vary between municipal supplies and independent reservoirs using distilled water, yet both systems are susceptible to contamination [5]. Even in units supplied with distilled water, biofilm formation has been documented [6,7]. Microbes can detach from these biofilms and enter a patient’s mouth via water irrigation or aerosol dispersion from handpieces [8]. Biofilm resilience complicates eradication, as standard disinfection methods often prove inadequate [9].

International guidelines differ in their microbial water safety thresholds. The U.S. CDC sets a limit of <500 CFU/mL for nonsurgical dental procedures [10], while the American Dental Association recommends a more stringent < 200 CFU/mL [11]. Kuwait’s Environmental Public Authority (EPA) adopts the strictest threshold of ≤100 CFU/mL and mandates the complete absence of *E. coli*, *P. aeruginosa*, and fecal streptococci in DUWLs [12,13]. These standards underscore the importance of regular testing and adherence to disinfection protocols. The prevalence of DUWL contamination has been reported as high as 96.7% globally, with levels fluctuating throughout the day depending on flushing practices and equipment design [14,15]. A 2022 local study at Kuwait University Dental Center found that basic adherence to manufacturer instructions was insufficient to reduce contamination unless supplemented with additional practices, such as flushing, draining, and disinfectant use [16].

Despite global and local recognition of these risks, no nationwide surveillance data exist for DUWL contamination across Kuwait’s six governorates. Such evidence is critical for informing public health policy, benchmarking Kuwait against international standards, and strengthening infection control protocols across dental services. This study therefore evaluates the prevalence of bacterial contamination in DUWLs across primary health care centers in Kuwait during 2022–2023, investigates variation by year, governorate, and outlet type, and examines predictors of non-compliance with microbial safety thresholds. By identifying contamination patterns and predictors, this study provides evidence to guide infection control protocols, improve disinfection strategies, and support water quality monitoring in Kuwait’s dental settings.

## 2. Materials and Methods

This study employed a retrospective cross-sectional design to evaluate bacteriological contamination in dental unit water lines (DUWLs) using secondary data from primary healthcare centers across Kuwait’s six governorates. Primary healthcare centers provide continuous, community-based care and represent the main entry point into the national health system.

The dataset was obtained from the Environmental Health Department within the Public Health Administration of the Ministry of Health and included all routine “water sample inspection request forms” submitted between January 2022 and December 2023. While the year of testing was consistently recorded, the month and exact date were frequently missing or unclear.

Water samples were collected by Ministry of Health inspectors and analyzed in accredited microbiology laboratories as part of Kuwait’s national environmental surveillance program. Inspectors maintain a comprehensive list of dental units in primary healthcare centers and apply a systematic random sampling approach, collecting samples monthly to ensure that all units are covered annually. By design, this process included both high- and low-volume clinics, thereby minimizing the risk of selection bias. In total, 3290 water testing records were available for analysis.

Each record contained the governorate, sampling date (when available), outlet type (cup filler, handpiece, or distillation unit), and bacteriological test result. In some cases, the inspection form included the name of the health center, which was used only to confirm the governorate of origin. Because this information was incomplete and inconsistently reported, the total number of unique centers or samples per center could not be reliably determined.

Samples were classified as bacteriologically permissible if the total plate count (TPC) was ≤100 colony-forming units per milliliter (CFU/mL), and no pathogens were detected. Samples were classified as contaminated (non-permissible) if the TPC exceeded 100 CFU/mL or if any of the following bacteria were present: coliforms, *Escherichia coli*, *Pseudomonas aeruginosa*, fecal streptococci, or other potential pathogens.

TPC values in the dataset ranged from 110 to 82,000 CFU/mL. Descriptive statistics, including the mean, standard deviation, median, and interquartile range, were calculated to summarize the distribution of TPC values. Laboratory-specific details on culture media and incubation conditions were not available in this secondary dataset, which we acknowledge as a study limitation.

All data were manually entered into Microsoft Excel for cleaning and validation before being analyzed in STATA version 17. Data entry was double-checked to minimize transcription errors, and consistency checks were performed prior to analysis. As the study included the complete population of 3290 water sample results, no additional sampling or power estimation was required.

The primary outcome variable was microbial contamination (contaminated vs. non-contaminated). Independent variables were the year of testing (2022 or 2023), governorate (Al-Asima, Al-Ahmadi, Al-Farwaniya, Hawalli, Al-Jahra, and Mubarak Alkabeer), and outlet type (handpiece, cup filler, or distillation unit). Descriptive statistics were presented as frequencies and percentages. Associations between contamination status and independent variables were tested using chi-square statistics. Variables with *p* < 0.10 in univariate analysis were entered into a multivariable logistic regression model. Adjusted associations were reported as odds ratios (ORs) with 95% confidence intervals (CIs), with statistical significance set at *p* < 0.05.

Because the dataset was anonymized and derived from routine surveillance forms, no individual patient information was included. Ethical approval was limited to secondary analysis of these surveillance data.

## 3. Results

### 3.1. Prevalence of Bacteriological Contamination (DUWLs)

A total of 3290 results were analyzed in our study (Table 1). The majority of samples were collected in 2023 (57.72%), primarily from the Capital Al-Asima governorate (27.9%) and the cup filler outlets (43.45%). Of these, 555 results (16.9%) showed microbial contamination. The highest proportion of contamination was observed in 2023 (19.9%), with Mubarak Alkabeer governorate showing 22.5% and the cup filler outlet 18.9%.

Samples with bacterial findings of TPC over 100 CFU/mL constituted 16.6%, with a range from 110 to 82,000 CFU/mL. Across all samples, the mean TPC was 659.1 CFU/mL, although the median was 0 (IQR: 0–0), reflecting the large number of sterile samples. Among contaminated samples only, the mean TPC was 3957 CFU/mL and the median was 2700 (IQR: 3100), providing a more representative summary of bacterial loads. *Pseudomonas aeruginosa* was detected in 1.3% of samples. This contrast between the near-zero median for all samples and the markedly elevated median among contaminated samples highlights the highly skewed distribution of TPC values, where most dental units were sterile but a minority exhibited heavy contamination.

**Figure 1:**
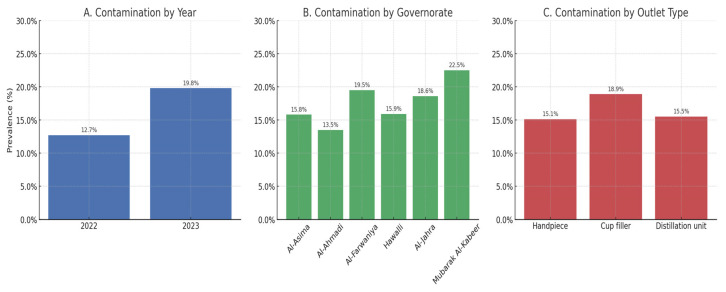
Prevalence of contaminated dental unit waterline samples by (**A**) year, (**B**) governorate, and (**C**) outlet type, Kuwait, 2022–2023.

A significant association was found between contamination and factors including the year of water testing, governorate, water sampling outlet, and the presence of *Pseudomonas aeruginosa* (*p* < 0.05). Figure 1 highlights these differences, showing the upward trend in contamination between 2022 and 2023, consistently higher prevalence in Mubarak Alkabeer governorate compared with other regions, and the greater likelihood of contamination from cup filler outlets relative to handpieces. In absolute terms, this difference translated into nearly one in five cup-filler samples being non-permissible, compared with about one in seven handpiece samples, underscoring the greater vulnerability of less frequently used outlets.

### 3.2. Multivariable Logistic Regression Model

The final adjusted multivariable logistic regression model (Table 2) indicated that, after accounting for the effects of other variables in the model, the odds of water bacteriological contamination in 2023 were 1.63 times that in 2022 (OR = 1.63, 95% CI: 1.34–1.98, *p* < 0.001). Among the six governorates, the odds of water bacteriological contamination in Mubarak Alkabeer were 1.42 times those in Al-Asima (OR = 1.42, 95% CI: 1.07–1.88). Additionally, water samples from the cup filler had 1.3 times the odds of bacterial contamination compared to samples from the handpiece (OR = 1.3, 95% CI: 1.3–1.62, *p* = 0.017). Although modest in magnitude, this elevated risk remained statistically significant after adjustment, suggesting that outlet type independently contributes to contamination risk beyond year and governorate effects.

## 4. Discussion

This study aimed to assess the prevalence of bacteriological contamination in dental unit waterlines (DUWLs) in primary healthcare centers across Kuwait during 2022 and 2023. It also sought to identify the most frequently contaminated water sampling outlets and explore variations across time, location, and outlet type. To our knowledge, this is the first nationwide effort in Kuwait to evaluate bacteriological DUWL safety using routine environmental inspection data from nearly all public dental departments in primary healthcare settings. The findings provide critical insights into potential microbial hazards in general dental care and emphasize the need for continuous surveillance and tailored infection control interventions to protect both patients and dental professionals. From a public health perspective, even moderate levels of DUWL contamination are important because they can expose large numbers of patients across primary care clinics. Since Kuwait’s PHC centers serve as the first point of contact for most dental care, ensuring microbiological safety in these settings has implications not only for individual patient safety but also for population-level infection control.

Our study found a bacterial contamination prevalence of 16.9%, based on Kuwait’s national and European C-100 standards, which require total plate counts (TPC) to remain below 100 CFU/mL. This level is notably lower than contamination rates reported in other regional and international settings [17]. For instance, a study in Saudi Arabia documented a prevalence of 42.2% [18], while systematic reviews covering Iran, Turkey, Iraq, and Jordan reported even higher rates [19]. In Iran, one meta-analysis found contamination rates as high as 69% using the same <100 CFU/mL threshold [20]. The relatively lower contamination rates observed in our study may reflect improved compliance with decontamination protocols or differing structural factors in Kuwait’s public dental infrastructure.

Elevated TPC was the predominant contributor to contamination, with 16.9% of samples exceeding the 100 CFU/mL threshold, and TPC values ranging from 110 to 82,000 CFU/mL. The distribution of TPC values was highly skewed due to the large proportion of sterile samples; therefore, descriptive statistics restricted to contaminated samples provide a more representative summary of microbial burden. These findings are lower than those reported in the Netherlands, where 61% of samples failed the EU benchmark [21], or in China, where 57.4% surpassed the threshold, with counts reaching 380,000 CFU/mL [22]. Differences across studies may reflect variations in study design, timing of sample collection, water line disinfection practices, or the specific sampling outlets tested. As for *Pseudomonas aeruginosa*, a known opportunistic pathogen in dental water systems, our study reported a low overall prevalence of 1.3%. Although this proportion is small, the clinical relevance remains important given the opportunistic nature of this pathogen. This compares favorably to other studies across the region and globally, where *P. aeruginosa* has been detected in 7.5% to 40% of DUWL samples [19,23,24,25]. These comparatively lower findings may point to effective chlorination or filtration in Kuwait’s systems or improved maintenance procedures in the public sector. Nonetheless, the variation in *P. aeruginosa* prevalence across countries underscores the need for uniform diagnostic methods and cross-comparable standards for DUWL safety assessment.

Contamination prevalence rose from 12.8% in 2022 to 19.8% in 2023—a 63% increase in odds of contamination. Similar upward trends were observed in Germany, where contamination rose from 50% to 55% between the same years [23]. These highlight the dynamic nature of microbial contamination and the importance of contextual factors, including maintenance quality, seasonal influences, and the operational burden following COVID-19 recovery, which may have strained maintenance routines and contributed to the observed rise in contamination. Internationally, a Canadian 11-year retrospective study found that 21% of dental handpiece samples failed the 500 CFU/mL standard, and 19% failed the stricter 100 CFU/mL limit, comparable to our findings. That study emphasized the importance of retesting following failures, as nearly half of initial failures remained above the acceptable threshold even after follow-up [26]. These results support the implementation of structured retesting protocols and stress the need for continuous quality monitoring. The observed increase between 2022 and 2023 may also reflect the gradual return of routine services and increased patient loads post-pandemic, which could have strained maintenance schedules. This trend suggests that contamination rates are sensitive to operational pressures and reinforces the importance of integrating DUWL monitoring into broader infection control audits.

Significant differences in contamination rates were also observed across Kuwait’s governorates. Although geographic variation in DUWL contamination has not been extensively studied in the literature, similar differences have been noted across dental specialties, with periodontics showing distinct microbial profiles [23]. Another study from Italy found that dental units relying on tank-based water systems had higher contamination (69%) than those connected to municipal water (56%) [27]. Such disparities may be related to unequal human resources, inconsistent training, or water supply differences between regions. These findings highlight the need to standardize DUWL maintenance procedures across all governorates and investigate regional resource gaps in dental infrastructure. Targeted interventions, such as standardized flushing protocols, scheduled shock disinfection, and improved staff training, could help reduce these regional disparities. Embedding DUWL surveillance within Kuwait’s existing environmental health monitoring system may also provide a cost-effective way to sustain improvements over time.

Our analysis revealed that bacterial contamination was 30% more likely in samples taken from cup-fillers compared to handpieces. This aligns with prior studies emphasizing the role of stagnant water in microbial proliferation [28]. Cup-fillers, being less frequently used, may foster water stagnation and biofilm formation, which increases bacterial loads. Even with the use of distilled water, contamination can persist if disinfection protocols are not rigorously maintained [29]. These findings reinforce the need for clearer guidance on routine outlet testing, line-specific disinfection schedules, and targeted protocols for less-used outlets. Further research is warranted to test the efficacy of anti-stagnation and automated flushing systems in reducing outlet-specific contamination risks.

### Limitations

This study has several limitations. First, comparisons with other studies are complicated by variations in microbial thresholds (100 CFU/mL in Kuwait vs. 200 CFU/mL by ADA and 500 CFU/mL by CDC), which limits regional benchmarking. Second, most published studies focus on private clinics or specialized hospitals, while our study was restricted to public primary care units that operate under different conditions. Third, the dataset lacked some contextual information, including water source, clinic workload, maintenance schedules, and consistent identifiers for individual centers, which prevented analysis at the center level. Fourth, the study covered only two years, limiting the ability to examine long-term or seasonal trends. Although the year of each sample was reliably recorded (as reports are archived annually), the month and day were not consistently documented, precluding seasonal analysis. Despite these constraints, the study provides a valuable nationwide snapshot of DUWL contamination in Kuwait and highlights priorities for surveillance, outlet-specific testing, and regional standardization of maintenance protocols.

## 5. Conclusions

This study demonstrates that the prevalence of bacteriological contamination in dental unit waterlines (DUWLs) across Kuwait’s primary healthcare centers is influenced by temporal, geographical, and outlet-specific factors. Notably, contamination increased in 2023 compared to 2022, suggesting a possible decline in maintenance efficiency or adherence to disinfection protocols post-pandemic. Geographic disparities were evident, particularly in Mubarak Alkabeer governorate, where contamination rates were substantially higher than in other regions. These variations highlight uneven implementation of infection control measures and underscore the necessity of standardizing protocols across governorates.

A significant finding was the elevated contamination rate in water samples collected from cup filler outlets compared to handpieces. This discrepancy may stem from the infrequent use of cup fillers, allowing for water stagnation and biofilm formation, and possibly from their omission in routine flushing or disinfection procedures. These insights call for enhanced disinfection efforts targeting less frequently used components and the incorporation of outlet-specific protocols in infection control guidelines.

Despite the overall lower prevalence of *Pseudomonas aeruginosa* compared to international benchmarks, its presence remains clinically relevant due to its opportunistic pathogenicity and resistance to disinfectants. Regular microbial monitoring, rigorous flushing procedures, and adherence to EPA and EU bacteriological standards (<100 CFU/mL) should be enforced to sustain water safety in dental care settings. In light of these findings, further research is warranted to investigate the underlying causes of regional discrepancies, particularly with respect to water supply sources, clinic workloads, and maintenance infrastructure. A broader surveillance framework and continuous quality improvement initiatives are essential to ensure consistent water quality and reinforce patient safety across all dental units in Kuwait’s public health system.

## Figures and Tables

**Table 1:** Descriptive characteristics of dental water results and total plate count (TPC) distribution by contamination status.

Characteristic	Total	Contamination Status		*p*-Value
		Uncontaminated	Contaminated	
	n (%)	n (%)	n (%)	
**Total**	3290 (100%)	2735 (83.1%)	555 (16.9%)	
**Year**				<0.001 ***
2022	1391 (42.3%)	1213 (87.2%)	178 (12.8%)	
2023	1899 (57.7%)	1522 (80.2%)	377 (19.8%)	
**Governorate**				0.001 **
Asima	919 (27.9%)	774 (84.2%)	145 (15.8%)	
Ahmadi	734 (22.3%)	635 (86.5%)	99 (13.5%)	
Farwaniya	394 (12.0%)	317 (80.5%)	77 (19.5%)	
Hawally	603 (18.3%)	507 (84.1%)	96 (15.9%)	
Jahra	156 (4.7%)	127 (81.4%)	29 (18.6%)	
Mubarak Alkabeer	484 (14.7%)	375 (77.5%)	109 (22.5%)	
**Water Sampling Outlet**				0.023 *
Handpiece	1050 (31.9%)	891 (84.9%)	159 (15.1%)	
Cup filler	1428 (43.4%)	1158 (81.1%)	270 (18.9%)	
Distillation unit	812 (24.7%)	686 (84.5%)	126 (15.5%)	
**Bacterial Finding**				<0.01 **
TPC ^1^	3290 (100%)	2742 (83.4%)	548 (16.6%)	
Coliform	3290 (100%)	0 (0.0%)	0 (0.0%)	
*E. coli*	3290 (100%)	0 (0.0%)	0 (0.0%)	
*P. aeruginosa*	3290 (100%)	3246 (98.7%)	44 (1.3%)	
*F. streptococci*	3290 (100%)	0 (0.0%)	0 (0.0%)	
Others	3290 (100%)	0 (0.0%)	0 (0.0%)	
**TPC descriptive statistics (CFU/mL)**				
Mean (all samples)	659.1	–	–	
Median (all samples, IQR)	0 (0–0)	–	–	
Mean (contaminated only)	3957.0	–	–	
Median (contaminated only, IQR)	2700 (1200–4300)	–	–	

* *p*-value < 0.05, ** *p*-value < 0.01, *** *p*-value < 0.001; *p*-value obtained by chi-square test. ^1^ Total Plate Count. TPC descriptive statistics include mean, median, and IQR. Values for ‘all samples’ reflect the skewed distribution due to the large proportion of sterile results, whereas ‘contaminated samples only’ provide a more representative summary of bacterial counts. Range = 110–82,000 CFU/mL.

**Table 2:** Adjusted logistic regression of contaminated dental water results.

Characteristic	OR	(95% CI)	*p*-Value
**Year**			
2022	Reference		
2023	1.6	(1.3, 2.0)	<0.001 ***
**Governorate**			
Asima	Reference		
Ahmadi	0.9	(0.7, 1.2)	0.332
Farwaniya	1.3	(1.0, 1.8)	0.104
Hawally	1.0	(0.8, 1.4)	0.877
Jahra	1.2	(0.8, 1.9)	0.369
Mubarak Alkabeer	1.4	(1.1, 1.9)	0.014 *
**Water Sampling Outlet**			
Handpiece	Reference		
Cup filler	1.3	(1.3, 1.6)	0.017 *
Distillation unit	1.1	(0.8, 1.4)	0.593

* *p*-value < 0.05, *** *p*-value < 0.001; *p*-value obtained by logistic regression test.

## Data Availability

Restrictions apply to the datasets: The datasets presented in this article is owned by the Ministry of Health, Kuwait.

## Abbreviations

The following abbreviations are used in this manuscript:

DUWLs	Dental Unit Water Lines, used in dental units to supply water to handpieces and cup fillers.
CFU	Colony Forming Unit, a standard used to measure bacterial concentration in water sample lab results.
IWR	Independent Water Reservoir, an attachable bottle to the dental chair.
WHO	World Health Organization
CDC	Center for Disease Control and Prevention
HPC	Hetero plate count
TPC	Total Plate Count
MOH	Ministry of Health
PHA	Public Health Administration
EHD	Environmental Health Department
ADA	American Dental Association
EPS	Extracellular polymeric substances
GCC	Gulf Cooperation Countries
Stata 17	A statistical software used to analyze data for descriptive and regression analysis.
